# F116. CAN INSIGHT LEAD TO REMISSION - FOR PATIENTS WITH SCHIZOPHRENIA?

**DOI:** 10.1093/schbul/sby017.647

**Published:** 2018-04-01

**Authors:** Maivor Olsson-Tall, Fredrik Hjärthag, Anna-Karin Olsson, Madeleine Johansson, Hawar Moradi, Lars Helldin

**Affiliations:** 1 NU Health Care Hospital; 2 Karlstad University

## Abstract

**Background:**

It is important to have extensive knowledge of the patients with schizophrenia, to provide the right support in outpatient care to create a good situation for the patient and prevent hospitalization. Lack of insight regarding the illness and symptoms might impair the patient’s ability to understand the illness, the treatment and relapses into psychotic episodes. Symptomatic remission is a well-established goal for treatment. If the core-symptoms do not affect their functions and the status is stable for at least six months, the patient is in remission. Previous research has shown an increased remission status from approximately 30% to more than 50% after using standardized remission criteria for patients with schizophrenia in Sweden. This study aims to investigate the relationship between both insight of symptoms and illness insight with cross-sectional remission.

**Methods:**

This is a cross-sectional study and the participants consisted of totally 289 patients with schizophrenia diagnosis. Of the participants 111 were women and 178 were men, with a mean age of 47 years (19–83 years old). Using semi-structured interviews and evidence-based assessment scales Remission Scale - Symptom (RS-S) and Psychosis Evaluation Tool for Common use by Caregivers (PECC) the data is collected. Cross tabulations were used to compare the distribution of the variables and the Pearson Chi-Square Tests for examine significant association.

**Results:**

Insight of symptom: The results show that from the patients who are not in remission, 69.5% are missing insight to symptoms, while 30.5% are having insight of symptoms. When it comes to the patients within remission, 59.0% have an insight of symptoms, while 41.0% are missing this sort of insight. The findings in the analysis with Chi-Square Tests examine independence indicated significant association between insight of symptoms and remission status (1, x2 = 22.17), p = <0,001, phi = -0.28.

Insight of illness: During the analysis of insight of illness, the results show that from the patients who are in remission, 61.9% possess insight of illness, compared to 38.1% of the patients lacking insight of illness. Concerning the insight of illness for the patients who are not in remission, 68.2% lack insight of illness, while 31.8% possess insight of illness. The Chi-Square Tests examine independence indicated significant association between insight of illness and remission status (1, x2 = 24.28), p = <0.001, phi = -0.29.

**Discussion:**

The results show that there is a relationship between insight of symptoms and illness with the cross-sectional symptomatic remission. However, the question is still open if remission is a consequence of insight or if the insight changes over time according to the activity of the illness. By following patients over time and monitoring the activity of symptoms including the state of remission and insight, it will probably be visualized if changes occur related to each other or independently. Also, whether the main focus for success is pharmaceutical treatment aiming for maximal symptom reduction or psychoeducational treatment to develop patients’ ability to understand their illness. Finally, whether insight after being established is a state or a treatment phenomenon? Further research to explore this issue is needed.

